# Smoking habits and alcohol use of patients with tuberculosis at Standerton Tuberculosis Specialised Hospital, Mpumalanga, South Africa

**DOI:** 10.4102/hsag.v24i0.1146

**Published:** 2019-10-08

**Authors:** Janke Wessels, Corinna M. Walsh, Mariette Nel

**Affiliations:** 1 Department of Nutrition and Dietetics, Faculty of Health Sciences University of the Free State, Bloemfontein, South Africa; 2 Department of Biostatistics, Faculty of Health Sciences University of the Free State, Bloemfontein, South Africa

**Keywords:** Tuberculosis, Smoking, Alcohol, Level of Education, Human Immunodeficiency Virus, Body Mass Index

## Abstract

**Background:**

A high prevalence of smoking and alcohol use has been reported in patients with tuberculosis (TB) by several researchers, even though these lifestyle habits have a negative impact on prognosis and treatment.

**Aim:**

To determine the smoking habits and alcohol use of patients with TB and TB/human immunodeficiency virus (HIV) co-infection, and how it is associated with gender, level of education and body mass index (BMI).

**Setting:**

The study was conducted at Standerton TB Specialised Hospital, Mpumalanga.

**Methods:**

A cross-sectional approach was applied. A structured interview was conducted by the researcher with each of the 100 hospitalised patients to obtain information about smoking habits, alcohol use and level of education. Weight and height were measured using standard techniques.

**Results:**

Almost six out of 10 participants (58%) indicated that they were former (44%) or current (14%) smokers. Nearly half (49%) reported that they used alcohol, with 25% drinking alcohol more than three times per week. Significantly more women than men were non-smokers (60.0% vs. 30.0%) and more men drank alcohol three times or more per week than women (36.7% vs. 7.5%). Participants who indicated that they were either former or current smokers had significantly lower levels of education than participants who were non-smokers (95% confidence interval [CI] [−26.7%; −2.6%] and [−39.9%; −1.0%] respectively).

**Conclusion:**

A high percentage of patients with TB and TB/HIV co-infection previously or currently smoked and used alcohol. Smoking and alcohol use are likely to have a negative impact on nutritional status and may further affect the prognosis of patients with TB.

## Introduction

Even though tuberculosis (TB) is a curable disease, in 2014 alone there were 1.5 million TB-related deaths worldwide. Approximately one-third of the world population, an estimation of more than 2 billion people, are infected with *Mycobacterium tuberculosis* (*Mtb*). Tuberculosis is an infectious disease caused by the *Mtb* organism entering the lungs (WHO 2015). Sites apart from the lungs that can also be infected include the lymph nodes, pleural cavities, pericardium, peritoneum, meninges, vertebral bodies and synovial tissue of other joints. Multi-organ involvement including the liver, spleen, lungs and bone marrow may also occur (Churchyard & Corbet 2008:438). Tuberculosis has impacts on health, nutrition, food security and socioeconomic development (WHO 2015).

Tobacco smoking has increased significantly over the past three decades, especially in developing countries. It is expected that smoking will cause about 10 million adult deaths in 2030 and most of the increased tobacco-related deaths will take place in Africa, Asia and South America (Wang & Shen 2009). It is estimated that up to 20% of TB cases globally are attributed to tobacco exposure (Gegia et al. 2015). Patients with TB who smoke have a nine times higher risk of mortality than patients with TB who have never smoked. In Taiwan, smoking accounts for more than one-third of TB-related deaths (Wen et al. 2010). In China, cigarette smoking has also been shown to be strongly associated with TB (Wang & Shen 2009). A study undertaken in Osaka city in Japan reported that the prevalence of smoking was significantly higher in patients with TB than in the general population (Matsumoto et al. 2012:547). According to the National Health and Nutrition Examination Survey (NHANES) 1999–2000 in the United States, smoking is a strong risk factor for latent TB infection in countries with a low TB prevalence (Horne et al. 2012). In a country such as India, where HIV infection is relatively uncommon, smoking and malnutrition are associated with a higher risk of acquiring TB (Bhargava et al. 2013). According to Horne et al. (2012), individuals with a higher prevalence of latent TB infection and an increased risk for progression to active TB disease might be identified through their smoking status. Thus, all smokers from high-risk populations should be considered for TB screening.

Cigarette smoking is associated with an increased lifetime risk for TB infection (Saad & Tirkey 2013). Both passive and active exposures to tobacco smoking have been shown to be associated with TB infection and progression to active TB (Saad & Tirkey 2013). It is more difficult to identify TB among smokers because smokers and patients with TB share a number of common clinical symptoms (Wen et al. 2010).

In addition to smoking, alcohol use has also been established to be a risk factor for acquiring TB, and continued use of alcohol and tobacco products once a person has contracted TB lowers the chances of successful treatment (Louwagie, Wouters & Ayo-Yusuf 2014:501). Alcohol is a relative risk factor for TB, especially in individuals who consume more than 40 g of alcohol per day (SADoH 2014). An earlier study by Coetzee, Yach and Joubert (1988) reported that the prevalence of TB was higher in households where alcohol use was considered to be problematic. More recently, Peltzer (2014) reported a high incidence of combined tobacco and alcohol use among patients with TB in primary care clinics in South Africa.

The China Health and Nutrition Survey concluded that smoking is associated with an increased risk of being underweight and a decreased risk of being overweight or obese (Wang 2015). PrayGod et al. (2013) found that patients with TB who smoked weighed less and were also more likely to have lower muscle mass and higher fat mass than those who did not smoke. Lower weight among smokers is thought to be because of a reduction in appetite and food intake as well as increased resting energy expenditure mediated by the effects of nicotine on body metabolism (PrayGod et al. 2013). Smoking is known to suppress the immune system, and when smoking is combined with HIV infection, the negative impact on TB development and progression is significant (Kolappan & Gopi 2002:965–966; Oni et al. 2012).

In view of the negative effects of smoking and alcohol use on patients with TB, the purpose of this study was to determine the smoking habits and alcohol use of patients with TB and TB/HIV co-infection at Standerton TB Specialised Hospital, Mpumalanga and how it is associated with gender, level of education and body mass index (BMI). This information may contribute to the identification of lifestyle habits that need to be taken into account in health and nutrition interventions, which might ultimately play a role in decreasing the impact of TB on the individual, the household and the wider community.

## Methodology

### Study design

A cross-sectional study was conducted.

### Target population and sampling

The study population included all patients aged between 20 and 65 years with TB and TB/HIV co-infection who gave informed consent to participate at Standerton TB Specialised Hospital, Mpumalanga, in wards 1 and 2 over a period of 1 month (21 July – 17 August 2015). Patients with additional diagnoses other than TB and TB/HIV co-infection, pregnant or lactating patients, and mentality or physically disabled patients were excluded from the study. The sample included 100 patients with TB and TB/HIV co-infection that met the inclusion criteria.

### Pilot study

A pilot study was conducted on the first five patients who met the inclusion criteria. The pilot study was undertaken to determine the feasibility of the methodology. As no changes were made to the questionnaire, the data of these participants were included in the main study.

### Variables and operational definitions

#### Smoking habits

Smoking habits were categorised as follows (Peltzer 2014; Saad & Tirkey 2013):

- Non-smoker: Patient who has never smoked.
- Former smoker: Patient who had smoked before, but who has stopped smoking for at least 3 months before entering the study.
- Current smoker: Patient who smokes at least one cigarette, pipe or cigar per day for at least 6 months prior to entering the study.

Patients who were former or current smokers were asked the question of how many times a day and for how many years they had been smoking.

#### Alcohol use

Alcohol consumption was categorised according to whether participants formerly or currently drank alcohol more than three times per week (Peltzer 2014; Saad & Tirkey 2013). An alcohol consumption of less than three times per week was considered low, while a consumption of three or more than three times a week was considered high (Saad & Tirkey 2013).

#### Level of education

Information related to sociodemographic status included gender and level of education.

#### Body mass index

Body mass index was calculated by dividing weight in kilograms (kg) by height in metre squared (m^2^). Body mass index was interpreted according to the World Health Organisation (WHO 2006) categories of BMI as underweight (< 18.5 kg/m^2^), normal weight (18.5 kg/m^2^ – 24.9 kg/m^2^) and overweight (> 25 kg/m^2^).

### Methods and techniques

#### Questionnaire

A questionnaire was designed by the researcher to obtain information regarding the smoking habits, alcohol use and level of education of patients with TB and TB/HIV co-infection. The researcher completed the questionnaire in a structured interview with each participant.

#### Weight

Weight was measured with a platform electronic scale (TCS-200-RT). As recommended by Lee and Nieman (2013:168), the participants were wearing minimal clothing (removed jacket, shoes and jewellery), standing still in the middle of the scale’s platform without touching anything and with the body weight equally distributed on both feet. Weight was recorded to the nearest 0.1 kg.

#### Height

Height was measured using a stadiometer (TCS-200-RT) with a vertical scale of 2 m and a sliding headpiece, to the nearest 0.5 cm. Height of the participants was measured without shoes. Participants stood with their heels together, arms to the side, legs straight, shoulders relaxed and head in the Frankfort horizontal plane (looking straight ahead). Heals, buttocks, scapulae and the back of the head were against the vertical surface of the stadiometer. Just before the measurement was taken, the participant inhaled deeply, held the breath and maintained an erect position while the sliding headpiece was lowered to the highest point of the head with enough pressure to compress the hair (Lee & Nieman 2013:167).

### Validity and reliability

Content validity was enhanced by ensuring that all data collected were directly related to the aim and objectives of the study. Reliability was enhanced by ensuring that all data were collected by a trained researcher using calibrated equipment and standardised techniques.

### Data collection

All eligible participants signed consent forms in their language of choice (English or IsiZulu) after the purpose and procedure of the study were explained to them by the researcher or a lay counsellor who spoke the native language. The information document was given to patients to provide them with all the relevant information regarding the study. Once informed consent had been obtained, participants were interviewed by the researcher and anthropometric measurements were taken in a private room. In addition to information on smoking habits and alcohol use, information on sociodemographic status, food security and nutrition status was also collected.

### Statistical analysis

Descriptive statistics, namely, frequencies and percentages for categorical data, and medians and percentiles for continuous data were calculated. Associations between variables were calculated and described by means of 95% confidence intervals (CI) for differences in percentages. All analyses were completed by the Department of Biostatistics at the University of the Free State.

### Ethical consideration

Ethical approval was obtained from the Provincial Health and Research Ethics Committee (PHREC) of Mpumalanga Department of Health (PHREC MP_2015RP38_556) and the Health Sciences Research Ethics Committee of the University of the Free State (ECUFS 56/2015).

## Results

The study sample included 100 participants (60 men and 40 women). The mean age of the sample was 39.2 years (age range 20.3–63.5 years). More than two-thirds of participants (68%) were HIV-positive, with HIV co-infection being slightly higher in women (70%) than in men (66.7%).

### Smoking habits

Table 1 illustrates the number of cigarettes, pipes or cigars smoked per day and the number of years that former or current smokers had smoked. Table 2 presents the smoking habits of male and female participants. About four out of 10 participants (42%) indicated that they were non-smokers, and 58% indicated they were former (44%) or current (14%) smokers. A significantly higher percentage of women (60%) than men (30%) were non-smokers (95% CI for the percentage difference [−10.2%; −47.0%]).

**TABLE 1:** Median smoking habits of former and current smokers (*n* = 58).

Question	Median	Range (min – max)
Number of cigarettes, pipes or cigars smoked per day	4	1–20
Number of years smoked	9	1–30

**TABLE 2:** Smoking habits (*n* = 100).

Smoking status	Male (*n* = 60)	Female (*n* = 40)	95% CI for the percentage difference
Non-smoker (*n* = 42)	30.0	60.0	(−47.0%; −10.2%)*
Former smoker (*n* = 44)	48.3	37.5	-
Current smoker (*n* = 14)	21.7	2.5	-

CI, confidence interval.

* Statistically significant difference.

Categories of level of education and BMI and associations with smoking habits are displayed in Table 3. Participants who stated that they were either former or current smokers had significantly lower levels of education than participants who were non-smokers. Although a higher percentage of participants who formerly or currently smoked had a BMI in the underweight category than those who had never smoked, the difference was not statistically significant.

**TABLE 3:** Level of education, body mass index categories and associations with smoking habits (*n* = 100).

Variables	Non-smoker (*n* = 42)	Former smoker (*n* = 44)	Current smoker (*n* = 14)	95% CI for the percentage difference
**Level of education (%)**				
No schooling	0.0	13.6	14.3	Non-former (−26.7%; −2.6%)* Non-current (−39.9%; −1.0%)* Former-current (−27.3%; 16.0%)
Less than Grade 9	33.3	38.6	35.7	
At least Grade 9	52.4	43.2	42.9	
Matric completed	9.5	4.6	7.1	
Tertiary education	4.8	0.0	0.0	
**BMI score (%)**				
< 18.5: Underweight	42.9	59.1	64.3	Non-former(−35.3%; 4.7%) Non-current(−45.2%; 8.1%) Former-current(−29.5%; 23.5%)
18.5–24.9: Normal weight	30.9	38.6	35.7	
25.0–29.9: Overweight	14.3	2.3	0.0	
> 29.9: Obese	11.9	0.0	0.0	

BMI, body mass index; CI, confidence interval.

* Statistically significant difference.

### Alcohol use

The alcohol use of male and female participants is presented in Table 4. More than half (51%) of the participants stated that they did not use alcohol. Almost half (49%) of the participants indicated that they used alcohol of which 25% indicated that they consumed alcohol more than three times per week and 24% indicated that they used alcohol less than three times per week. Men consumed alcohol (three or more times per week) significantly more often than women (95% CI for the percentage difference [12.6%; 42.7%]).

**TABLE 4:** Alcohol use (*n* = 100).

Variable	Male (*n* = 60)	Female (*n* = 40)	95% CI for the percentage difference
Drink alcohol three or more times per week (*n* = 25)	36.7	7.5	(12.6%; 42.7%)*
Drink alcohol less than three times per week (*n* = 24)	31.7	12.5	-
Do not drink alcohol (*n* = 51)	31.7	80.0	-

CI, confidence interval.

* Statistically significant difference.

The associations between alcohol use and level of education and BMI scores are presented in Table 5. There were no statistically significant differences between the percentage of participants who did or did not drink alcohol in terms of level of education and BMI scores.

**TABLE 5:** Level of education, body mass index categories and associations with alcohol use (*n* = 100).

Variables	Used to drink alcohol three or more times per week (*n* = 25)	Used to drink alcohol less than three times per week (*n* = 24)	Do not drink alcohol (*n* = 51)	95% CI for the percentage difference
**Level of education (%)**				
No schooling	4.0	8.3	9.8	> 3/week – < 3/week (−22.2%; 12.3%) > 3/week – none (−17.5%; 10.7%) < 3/week – none (−14.2%; 16.9%)
Less than Grade 9	52.0	29.2	31.4	
At least Grade 9	36.0	54.2	49.0	
Matric completed	4.0	8.3	7.8	
Tertiary education	4.0	0.0	2.0	
**BMI score (%)**				
< 18.5: Underweight	56.0	62.5	47.1	> 3/week – < 3/week (−31.5%; 19.8%) > 3/week – none (−14.3%; 30.6%) < 3/week – none (−8.5%; 36.3%)
18.5–24.9: Normal weight	40.0	37.5	31.4	
25.0–29.9: Overweight	4.0	0.0	11.8	
> 29.9: Obese	0.0	0.0	9.8	

BMI, body mass index; CI, confidence interval.

* Statistically significant difference.

### Combined smoking habits and alcohol use

Combined smoking habits and alcohol use are shown in Table 6. Only 35 participants indicated that they did not drink alcohol and were non-smokers. Heavy drinking habits (of more than three times per day) together with current smoking were noted in eight participants.

**TABLE 6:** Combined smoking habits and alcohol use (*n* = 100).

% (*n*)	Used to drink alcohol three or more times per week (*n* = 25)		Used to drink alcohol less than three times per week (*n* = 24)		Do not drink alcohol (*n* = 51)	
	*n*	%	*n*	%	*n*	%
Non-smoker (*n* = 42)	4.8	2	11.9	5	83.3	35
Former smoker (*n* = 44)	34.1	15	34.1	15	31.8	14
Current smoker (*n* = 14)	57.1	8	28.6	4	12.3	2

## Discussion

The results of this study indicated that a high percentage of patients with TB and TB/HIV co-infection smoked and used alcohol. The smoking habits and alcohol use of patients with TB have been reported in a number of other studies (Awan, Waqas & Aslam 2012; Biranvand et al. 2012; Coetzee et al. 1988; Feingold 1976; Horne et al. 2012; Kolappan & Gopi 2002; Lombardo, Swart & Visser 2012; Louwagie et al. 2014; Matsumoto et al. 2012; Peltzer 2014; PrayGod et al. 2013; Saad & Tirkey 2013; Singh et al. 2013; Wang & Shen 2009; Wen et al. 2010) all of whom found that these unhealthy lifestyle habits were very prevalent in patients with TB.

A study among patients with TB in Pakistan reported that 42.5% of participants smoked and 1.7% used alcohol at the time that the study was undertaken (Awan et al. 2012). In newly diagnosed patients with TB in Tanzania, 24.4% were current smokers and 54.2% reported that they consumed alcohol (PrayGod et al. 2013). Similarly, smoking was prevalent in 26.4% and alcohol use in 50.7% of patients with TB in Uganda (Kirenga et al. 2015). Ciegieski, Arab and Cornoni-Huntley (2012) reported that 79% of current smokers and 23.5% of persons who consumed more than seven alcoholic drinks per week developed TB later in life, listing both smoking and alcohol consumption as risk factors for the development of TB in the United States. More than 40 years ago, Feingold (1976) conducted a hospital-based study and found a 49% prevalence of alcoholism in patients newly diagnosed with TB in Georgia. Nearly three decades ago, Coetzee et al. (1988) found that frequent alcohol consumption was a risk factor for the development of TB in households from Mamre in Cape Town.

A study of 1005 male patients with TB in Tshwane Metropolitan Municipality, South Africa, reported that 37.6% of participants smoked and 27.3% were alcohol dependent (Louwagie et al. 2014). Similar results were found in the current study where 58% of participants were either former (44%) or current (14%) smokers and 49% of participants used alcohol. This study was conducted amongst hospitalised patients, and despite this, 14% participants indicated that they were currently smoking. Louwagie et al. (2014) reported even higher levels of smoking and alcohol use in patients with TB in South Africa, with 79% of participants in their study currently smoking and 23.5% consuming more than seven alcoholic drinks per week. Another study undertaken in South Africa amongst a large sample of 4900 patients with TB reported that 10.1% of participants (15.5% men and 3.4% women) smoked and used alcohol simultaneously (Peltzer 2014). In the current study, similar results were found with 8% of participants previously drinking alcohol heavily (i.e. used alcohol more than three times per week) together with current smoking. Smoking may also increase the risk of those living with patients with TB to TB infection because of passive smoking exposure (Matsumoto et al. 2012:547).

Smoking and alcohol use is more common in men with TB. The current study confirmed that significantly more men smoked and drank alcohol (three or more times per week) than women. Similar findings have been reported by other researchers (Kolappan & Gopi 2002; Singh et al. 2013). Smoking prevalence amongst adult men with TB in India was two to four times higher than amongst women and a study conducted by Kolappan and Gopi (2002) found a positive association between tobacco smoking and having pulmonary TB in Indian men (odds ratio [OR] = 2.48). A study in Iran amongst 183 patients with TB also found that men were significantly more likely to be smokers than women (OR = 12.4) (Biranvand et al. 2012). Furthermore, a study that included a large national sample of adult patients with a history of TB in Cambodia reported that TB infection was more common in men who smoked manufactured cigarettes and in those who were the heaviest smokers (more than one pack per day, more than 30 packs per month) (Singh et al. 2013). This was also confirmed in an Indian study where both cumulative smoking years and number of cigarettes smoked were associated with a significantly increased risk of TB (Saad & Tirkey 2013).

Other factors that are strongly associated with smoking and alcohol use have been identified by several researchers. Louwagie et al. (2014) concluded that drug, tobacco and alcohol use are closely related to poverty in patients with TB in South Africa. Saad and Tirkey (2013:340) reported that patients with TB who smoked were more likely to be older adults with lower levels of education and a history of drinking alcohol. Similarly, male gender, a lower level of education and higher levels of poverty were associated with simultaneous alcohol and tobacco use, as well as with alcohol or tobacco use amongst patients with TB in South Africa (Peltzer 2014). An analysis of 14 countries with a high TB burden found that smoking, alcohol consumption and BMI <18.5 kg/m² were independently associated with TB (PrayGod et al. 2013).

In the current study, a statistically significant difference was found between the levels of education of non-smokers and smokers, with smokers having a lower level of education (no schooling completed and less than Grade 9) than non-smokers. Although a high percentage of participants that were underweight (according to their BMI scores) smoked and used alcohol (42.9% vs. 56%), the difference was not statistically significant.

Smoking and alcohol status were based on the patient’s self-report rather than on the detection of nicotine or alcohol levels. Therefore, we acknowledge that the results of this single-centre study may not be generalised to all patients with TB and TB/HIV co-infection.

## Conclusion and recommendations

The high prevalence of smoking and alcohol use and associations with level of education that were identified amongst patients with TB and TB/HIV co-infection in the current study may impact treatment outcomes. It is recommended that guidelines on smoking and alcohol use should be incorporated into the National TB control plan and should also be included in Directly Observed Therapy Short-course (DOTS) interventions (Wang & Shen 2009). Stricter health policies on smoking and heavy drinking could be implemented in populations where TB is a major problem in order to improve health and quality of life (Awan et al. 2012:331; PrayGod et al. 2013).

The findings of this study further highlight the viscous cycle that exists, with poverty promoting the spread of TB and HIV and TB and HIV contributing to continuing poverty. Interventions aimed at improving outcomes in patients with TB and TB/HIV co-infection should ultimately include a more holistic approach to address the underlying determinants that are deeply rooted in poverty.
